# Depressive and anxiety symptoms among adults in Germany: Results from the RKI Panel ‘Health in Germany’ 2024

**DOI:** 10.25646/13573

**Published:** 2025-12-05

**Authors:** Lena Walther, Felicitas Vogelgesang, Angelika Schaffrath Rosario, Christina Kersjes, Julia Thom, Diana Peitz, Florian Beese, Heike Hölling, Elvira Mauz

**Affiliations:** Robert Koch Institute, Department of Epidemiology and Health Monitoring, Berlin, Germany

**Keywords:** Adults, Women, PHQ, Depression, Mental health, Prevalence, Anxiety, Education, Panel, Germany

## Abstract

**Background:**

Telephone surveys show a decline in the mental health of adults in Germany between 2020 and 2023. For 2024, results from the Robert Koch Institute’s new panel ‘Health in Germany’ on depressive and anxiety symptoms are presented and contextualised within existing time series.

**Methods:**

Using data from n = 27,102 participants surveyed online or on paper, prevalences were estimated and subgroup differences were examined. Trends for 2014 – 2024 were modelled taking into account methodological discontinuities.

**Results:**

In 2024, an estimated 22 % of adults showed depressive symptoms (PHQ-9 ≥ 10) and 14 % showed anxiety symptoms (GAD-7 ≥ 10). 8 % of adults had moderately severe to severe depressive or anxiety symptoms (PHQ-9/GAD-7 ≥ 15). Women, younger adults and people with low or medium levels of education were more frequently affected. The burden was particularly high among young women: 47 % showed depressive or anxiety symptoms. The figures for 2024 are significantly higher than those for the previous year; however, these differences appear to be largely due to a change in methodology. Whether the symptom rise observed from 2020 to 2023 continued in 2024 cannot be conclusively assessed owing to this methodological transition.

**Conclusions:**

There continues to be a high demand for measures to promote mental health in the population. The establishment of the RKI Panel in the coming years will enable methodologically consistent monitoring of depressive and anxiety symptoms in the future, which is a key prerequisite for the reliable assessment of trends.

## 1. Introduction

Mental health is an essential component of public health [1]. Depression and anxiety disorders in particular are widespread [2] and associated with a high individual and societal burden of disease [3–5]. These disorders, which are often recurrent or chronic [6], are strongly influenced by stressful life events or circumstances [7] and can severely limit the quality of life, functioning and participation of those affected in various areas of life [8, 9]. Diagnoses of depression and anxiety disorders account for a high number of absences among employees [10]. They are also associated with considerable healthcare costs [11]. Symptoms of depression and anxiety disorders are classified as internalising mental health problems [12, 13] and often occur together [14, 15]. This can lead to an even higher burden of disease and an increased risk of chronicity or recurrence, reduced treatability and an increased risk of suicidal thoughts and actions [6, 16]. Comorbidities with other mental disorders and physical illnesses are also common [14, 15, 17] and associated with increased mortality [14, 18, 19]. The association between depressive and anxiety disorders with suicidality also contributes to an increased risk of mortality [18].

Depressive and anxiety disorders are monitored as part of mental health surveillance [20] within the Robert Koch Institute’s (RKI) surveillance programme for non-communicable diseases (NCD surveillance). Monitoring the development of depression and anxiety disorders has so far been based on the analysis of statutory health insurance claims data on diagnoses in outpatient settings [21, 52] and on the observation of symptoms in the population using screening questionnaires in survey studies [22, 23].

Depressive symptoms were observed from 2014 to 2023 in several waves of the RKI study *German Health Update* (GEDA) among adults aged 18 and over. The 2014/2015 survey was based on a written online and paper survey in a sample randomly drawn from local authorities’ registers (registry sample) [24]. From 2019 onwards, data was collected via telephone interviews in a dual-frame telephone sample (i.e. landline and mobile phone) [25]. While the prevalence of depressive symptoms remained largely stable until 2020, there was a significant increase between 2020 and 2023 [26]. This trend of initial stability followed by an increase from 2020 onwards was also observed for more severe symptoms and across subgroups by gender, age and education.

Anxiety symptoms, assessed in the telephone surveys from 2021 onwards using the Generalised Anxiety Disorder-2 (GAD-2) short screener, also showed an increase across all subgroups until 2023 [27, 28]. In line with national and international studies (see [29–31] ), women were generally more affected by anxiety and depression symptoms than men, younger adults more than older adults, and people with low levels of education more often than those with high levels of education [22, 26–28, 32].

This article reports on current findings based on data from the new RKI Panel ‘Health in Germany’ for the year 2024. Like GEDA 2014/2015, the RKI Panel surveys a registry sample using written questionnaires (paper/online) [33]. Based on the long versions of the screening instruments for depressive symptoms (Patient Health Questionnaire-9, PHQ-9) and anxiety symptoms (GAD-7), the following results are reported, also stratified by gender, age and education: 1) The prevalence of depressive or anxiety symptoms according to validated screening thresholds, 2) the prevalence of more severe depressive or anxiety symptoms according to higher thresholds. In view of frequent comorbidity between the symptom patterns, 3) the prevalence of depressive or anxiety symptoms as well as more severe depressive or more severe anxiety symptoms is also considered. Risk groups by gender, age and education are also examined, controlling for the respective other sociodemographic characteristics. Finally, the results from 2024 are cotextualised within existing time series based on previous RKI health monitoring studies. These analyses take into account the change in study design, which may have a significant impact on prevalence estimates [34].

**Key messages**

► An estimated one in four adults (25 %) showed symptoms of depression or anxiety in 2024.
► 8 % of adults had moderately severe to severe depressive or anxiety symptoms.
► Women, younger adults and people with low and medium levels of education were more likely to experience depressive or anxiety symptoms than their respective comparison groups.
► Young women (aged 18 to 29) were most at risk, with almost half (47 %) exhibiting symptoms of depression or anxiety.
► Given the change in the survey methodology in 2024, it is neither possible to rule out nor to confirm a continuation of the increase in symptom prevalence observed between 2020 and 2023.

## 2. Methods

### 2.1 Study design and sample

#### RKI Panel ‘Health in Germany’

The panel Health in Germany was established in 2024 beginning with a recruitment study. The sampling was based on a two-stage random selection process: 359 primary sampling units, known as sample points, were randomly selected from all municipalities in Germany, taking into account the regional structure (first selection stage). In the second selection stage, addresses were randomly drawn from the address registers of the respective residents’ registration offices for each sample point, stratified by age group. The selected individuals were invited to participate in a short survey and asked to consent to participation in future surveys as part of the panel [33]. A mixed-mode approach was used, which allowed for both online participation and written participation by post [35].

For the 2024 annual survey, the RKI Panel comprised 46,863 registered participants aged 18 and over (data set version 5): 24,881 women, 21,856 men, 126 persons with other gender identities. In 2024, they were invited to participate in health surveys at three points in time (sub-waves) at intervals of approximately two months. Data collection began in May 2024 with the first sub-wave and was completed in early January 2025 with the third sub-wave. There was a total of four questionnaires on different topics [36]. Registered panellists received one of the four questionnaires in each of the three sub-waves in a predefined rotation so that each participant received three of the four questionnaires in 2024. The repeat participation rate (proportion of participants in relation to the number of registered panellists) in the individual waves was between 75 % and 81 % in accordance with the standards of the American Association for Public Opinion Research (AAPOR [37]). The cumulative response rate for all those invited to participate in the recruitment study (approx. 167,000 persons) was 22 %. A detailed description of the methodology and response rate (also stratified by age and gender) can be found elsewhere [38]. All mental health indicators were included in questionnaire C (see Infobox). The sample characteristics for this questionnaire can be found in the appendix (Annex Table 1).

#### German Health Update (GEDA) surveys

The results from 2024 were contextualised within the previous time series using data from telephone health surveys from various waves of the RKI GEDA studies from 2014/2015, 2019/2020, 2022 and 2023, as well as from the GEDA 2024 survey, which was conducted in parallel with the first four data collection months of the RKI Panel in the summer of 2024 for a methodological study. With the exception of GEDA 2014/2015-European Health Interview Survey (EHIS), all surveys were telephone interviews based on telephone samples (dual-frame samples based on landline and mobile numbers). In GEDA 2014/2015-EHIS, the sample was drawn from the population register and there was a sequential written mixed-mode design, in which the online questionnaire was the default and a paper questionnaire was only offered together with a participation reminder. The design of GEDA 2014/2015-EHIS is thus similar to that of the RKI Panel, with the only difference being that the panel offered a simultaneous mixed-mode design directly to people aged 70 and over. Accordingly, two types of studies were defined for the analyses. Studies with a registry sample and a written mixed-mode design (i.e. RKI Panel 2024 and GEDA 2014/2015-EHIS) were assigned to study type 1; the GEDA studies based on a telephone sample and telephone interviews were assigned to study type 2. Only data from persons aged 18 and older were included in the analysis.

### 2.2 Weighting

In order to correct for distortions due to selective participation and deviations of the sample from the population structure, a multi-stage sample weight was calculated for the RKI Panel 2024. It first accounts for the sampling weight of the initial recruitment study. In addition, dropout weights are calculated based on the recruitment study data to counteract selective participation in the repeated sub-waves. Finally, adjustment to population figures as of December 31, 2023, and the 2021 Microcensus were calculated. Age, gender, municipality size class, education (Comparative Analysis of Social Mobility in Industrial Nations, CASMIN) [39] and household size are taken into account in the adjustment weightings. The weighting was calculated separately for each questionnaire variant; the weights are defined for ages 18 and above. A detailed methodological description will follow in a separate article [40]. The weighting for trend modelling is described in detail elsewhere [41].

**RKI Panel ‘Health in Germany’ 2024**

**Data holder:** Robert Koch Institute

**Objectives:** To provide comprehensive data on the health status, health-related behaviour and health care of the population in Germany, with the future possibility of longitudinal comparisons and analysis of trends over time

**Study design:** Panel study with a mixed-mode approach (online and written-postal participation)

**Population:** German-speaking population aged 18 and over in private households with main residence in Germany

**Sample:** Probabilistic/representative sample of the Health in Germany panel infrastructure

**Participants in the 2024 annual wave:** A total of 41,376 of the persons registered in the panel took part in at least one of the three sub-waves in 2024.

Questionnaire A: 14,759 women, 12,374 men, 66 persons with other gender identities

Questionnaire B: 15,828 women, 12,258 men, 61 persons with other gender identities

Questionnaire C: 14,709 women, 12,329 men, 64 persons with other gender identities

Questionnaire D: 14,872 women, 12,368 men, 66 persons with other gender identities

**Data collection:**

1st sub-wave: 28.05.2024 to 05.08.2024

2nd sub-wave: 12.08.2024 to 14.10.2024

3rd sub-wave: 28.10.2024 to 06.01.2025

More information at www.rki.de/panel-en

### 2.3 Indicators

#### Depressive symptoms

Depressive symptoms were measured using the PHQ-9 [42] self-report questionnaire. The instrument covers the nine diagnostic criteria for depression according to the Diagnostic and Statistical Manual of Mental Disorders IV (DSM-IV, criteria for ‘major depression’): Depressed mood, loss of interest, sleep disturbances, fatigue, appetite changes, low self-worth, concentration problems, psychomotor retardation or agitation, and thoughts of death or suicide. Respondents are asked to indicate the frequency with which they have been affected by each of these symptoms in the last two weeks (‘not at all’ (value 0), ‘several days’ (value 1), ‘more than half the days’ (value 2) and ‘nearly every day’ (value 3)), resulting in total scores between 0 and 27. The recommended screening threshold of 10 for detecting possible depression has been repeatedly validated on the basis of diagnoses from comprehensive clinical interviews [43]. In addition to this binary outcome, the PHQ-9 is also divided into five severity levels: no to minimal symptoms (0 – 4), mild symptoms (5 – 9), moderate symptoms (10 – 14), moderately severe symptoms (15 – 19), severe symptoms (≥ 20) [42]. Accordingly, a PHQ-9 scale total score of ≥ 10 is assumed to indicate depressive symptoms and a PHQ-9 scale total score of ≥ 15 is assumed to indicate moderately severe to severe depressive symptoms. For trend modelling, the PHQ-8 [44], which is one item shorter (thoughts of death/suicidal thoughts), is used with the same threshold value [43] for comparability over time (see Contextualisation within existing time series).

#### Anxiety symptoms

Anxiety symptoms were assessed using the self-report instrument GAD-7 [45]. Its seven items cover the three areas of the DSM-IV criteria for generalised anxiety disorder: A - excessive anxiety and worry, B - difficulty controlling this anxiety and worry, C - accompanying symptoms such as restlessness and irritability. The overarching question and response categories are identical to those of the PHQ-9, resulting in total scores between 0 and 21 for seven items. The recommended screening threshold of 10 for detecting a possible generalised anxiety disorder [45] has been validated on the basis of diagnoses from comprehensive clinical interviews, whereby this threshold also achieves acceptable accuracy in detecting the possible presence of other anxiety disorders (such as panic disorder or social phobia) [46]. Thresholds of 5, 10 and 15 have also been recommended to demarcate different severity levels [45]. Accordingly, a scale sum value of GAD-7 ≥ 10 is assumed to indicate anxiety symptoms and a scale sum value of GAD-7 ≥ 15 is assumed to indicate more severe anxiety symptoms.

### 2.4 Stratification

To describe gender differences, the RKI Panel includes question both on gender assigned at birth and gender identity (including open-ended response options). The analyses by gender include individuals who identify as female or male. Gender-diverse individuals who do not fit into these categories are not reported separately in the analyses by gender due to the small number of cases (n = 64). Participants who did not provide information on their gender identity were categorised according to their reported gender assigned at birth. Five age groups were formed: 18 – 29 years, 30 – 44 years, 45 – 64 years, 65 – 79 years, and 80 years and older. Participants’ level of education was classified according to the CASMIN system, which groups formal school and vocational education into three categories: low (primary to lower secondary education), medium (middle/upper secondary education) and high (tertiary education) [39].

### 2.5 Statistical methods

#### Depressive and anxiety symptoms in the RKI Panel 2024

Descriptive analyses were performed taking into account key sociodemographic characteristics. Weighted proportions with PHQ-9 or GAD-7 ≥ 10 and PHQ-9 or GAD-7 ≥ 15 were estimated with 95 % confidence intervals stratified according to 1) gender, age and education, 2) gender and age, and 3) gender and education. In order to statistically assess group differences while controlling for the respective other two socio-demographic characteristics, robust Poisson regressions were calculated for depressive and anxiety symptoms as dependent variables and gender, age and education as independent variables. Statistical significance was set at p < 0.05. Possible interaction effects between gender and age, as well as between gender and education, were examined by including the corresponding interaction terms in the Poisson regression models. In order to limit the number of statistical tests, interactions between individual age or education groups and gender were only examined if they proved to be significant overall in an omnibus test (Wald test, significance criterion also p < 0.05). Missing values were deleted on a case-by-case basis, excluding 593 observations for the PHQ-9 and 385 observations for the GAD-7. 49 entries were missing for education. Prevalence estimates and Poisson regressions were performed using survey procedures in Stata/SE 17.0, taking into account weighting and data collection within municipalities.

#### Contextualisation within existing time series

Results from the RKI Panel 2024 are incorporated into existing time series from the RKI’s health monitoring accounting for changes in methodology from a registry sample and a written survey (paper/online) (GEDA 2014/2015-EHIS, study type 1) to a telephone survey in a dual-frame telephone sample (GEDA 2019 – 2024, study type 2) back to study type 1 (RKI Panel 2024). For this purpose, the PHQ-8 collected since GEDA 2014/2015 is used instead of the PHQ-9, which has only been used in recent years. The aim of the analysis is to model how the prevalence of depressive symptoms (PHQ-8 ≥ 10) would have developed had survey methodology remained consistent over time. To this end, the possible impact of study methodology is quantified using a model comparing the time trend between the two type 1 studies with the trend between the four type 2 studies. Importantly, data from both study types is available for an overlapping timeframe in 2024 (RKI Panel 2024 and GEDA 2024). The models are based on the central assumption that the effect of survey method is constant over time. To estimate the trend and differences by methodology, a logistic regression model was constructed for the total population and stratified by gender, age and education, with the dependent variable PHQ-8 ≥ 10 and the independent variables survey date (middle survey date per survey) and study design (binary variable: study type 1; study type 2). In order to estimate the trend over time as accurately as possible based on the available data, various trend types (linear, quadratic, flexible modelling using splines) were tested within the regression models for the time variable. A detailed description of the methodology used can be found in an article specifically on the investigation of the change in methodology between the GEDA studies and the RKI Panel [41]. In this article, trend model graphs are used to assess developments and effects of method. In addition, the magnitude of these effects, including 95 % confidence intervals for the total population and by gender, age and education (with p-values from Wald tests for group differences), is reported in order to assess prevalence differences between 2023 and 2024. The effect of method is quantified as the difference between the prevalence predicted by the model for study type 1 minus the corresponding prevalence for study type 2. The calculations were performed using survey procedures in SAS 9.4 (SAS Institute, Cary, NC, USA).

## 3. Results

### 3.1 Depressive symptoms and anxiety symptoms in adults in Germany in 2024

According to data from the RKI Panel 2024, 21.9 % of adults in Germany experienced depressive symptoms in 2024 (Table 1). Approximately one in three affected individuals – corresponding to 7.1 % of the total population – had moderately severe to severe symptoms. Anxiety symptoms were present in 14.3 % of adults; of these, about one in three to four (4.1 % of the total population) were more severely affected.

Overall, 25.1 % of adults exhibited depressive or anxiety symptoms, indicating a significant overlap between the affected groups. 11.1 % (confidence interval (CI): 10.5 % – 11.7 %]) (not included in Table 1) of the adult population had both depressive and anxiety symptoms. A total of 8.4 % of adults were found to have more severe depressive or anxiety symptoms.

Women were more likely to show depressive symptoms or anxiety symptoms than men. Similar relative risks (RR) for gender were found for the two symptom types after adjusting for age and education: compared to men, women had a 1.3-fold risk of depressive symptoms and a 1.5-fold risk of anxiety symptoms (Annex Table 2 and Annex Table 7). Regarding more severe symptoms, the differences by gender were slightly more pronounced for anxiety symptoms (RR for women compared to men: 2.0; Annex Table 8) than for depressive symptoms (RR for women: 1.4; Annex Table 5). Overall, one in ten women (9.8 %) were found to have been affected by more severe depressive or anxiety symptoms.

A comparison of age groups shows that the prevalence of both depressive and anxiety symptoms decreases with age up to the age of 65 – 79. For those aged 80 and older, the prevalence is higher than in the 65 – 79 age group, but not higher than in younger age groups: after controlling for gender and education, the proportion of people affected is significantly lower among the elderly than in the reference group of 45 – 64-year-olds (Annex Table 2, Annex Table 5, Annex Table 7, Annex Table 8).

Young women aged 18 to 29 appear to be particularly affected: 41.7 % of this group showed depressive symptoms, with more than a third (16.1 %) experiencing severe depressive symptoms (see Table 2). Overall, almost half of young women (47.0 %) showed depressive or anxiety symptoms, compared to one third of young men (31.3 %). In the case of depressive symptoms (PHQ-9 ≥ 10 and PHQ-9 ≥ 15) but not anxiety symptoms, Wald tests indicate a significant interaction between gender and age (p = 0.0114; p = 0.0393; Annex Table 3, Annex Table 6; anxiety symptoms: p = 0.2343; p = 0.1164).

There is a significantly lower prevalence of depressive and anxiety symptoms in the high education group compared to the low and medium education groups. After adjustment, an education gradient becomes apparent (Annex Table 2, Annex Table 5, Annex Table 7, Annex Table 8): among people of the same gender and age, those in the low education group are also more frequently affected than those in the medium education group. Adults in the low education group have almost twice the risk of depressive symptoms as those in the high education group controlling for gender and age (RR: 1.8). The risk of more severe depressive symptoms is almost three times as high for adults in the low education group than for adults in the high education group (RR: 2.7). The differences are slightly smaller for anxiety symptoms (RR low vs. high education group: 1.6 for anxiety symptoms and 2.0 for more severe symptoms). Men in the low education group are affected by depressive and anxiety symptoms to a similar extent as women in the low education group, whereas in the middle and high education groups, there are clear gender differences to the detriment of women. For depressive symptoms (PHQ-9 ≥ 10), this is reflected in a significant interaction between gender and education with adjustment for age (Wald test p = 0.0352; Annex Table 4). There is no statistically significant interaction between gender and education for anxiety symptoms (GAD-7 ≥ 10: p = 0.0843) or more severe symptoms (PHQ-9 ≥ 15: p = 0.8914; GAD-7 ≥ 15: p = 0.6706).

### 3.2 Contextualisation of prevalence estimates for 2024 within previous time series based on RKI studies

The prevalence of depressive symptoms in 2024 can be regarded in the context of previous time series using the PHQ-8, which is one item shorter than the PHQ-9 (see also 2.5 Statistical methods). Prevalence estimates are slightly lower using the PHQ-8 than the PHQ-9 (Annex Table 9).

**Figure 1:**
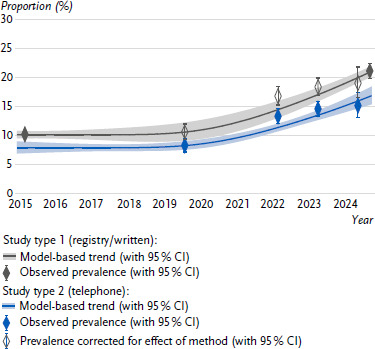
Depressive symptoms (PHQ-8 ≥ 10) 2014/2015 – 2024. Source: GEDA 2014/2015-EHIS, GEDA 2019/2020-EHIS, GEDA 2022, GEDA 2023, GEDA 2024, RKI Panel 2024

Figure 1 shows the estimate for the prevalence of depressive symptoms (PHQ-8 ≥ 10) in the total adult population based on data from the RKI Panel 2024 (grey diamond) in the context of the estimates reported previously. These come from studies with dual-frame telephone sampling and telephone interviews (study type 2, blue diamonds for GEDA 2019/2020-EHIS to 2024) as well as from studies with registry samples and self-administered questionnaires (paper/online) (study type 1, grey diamond for GEDA 2014/2015-EHIS). Diamonds with grey borders show the estimates from study type 2 adjusted to study type 1, i.e. corrected by the estimated effect of study design differences. These estimates model how high the prevalence would have been for 2019 to 2023 if data had been collected using the same method as the new RKI Panel. Modelled time trends for study type 1 (grey) and study type 2 (blue) are also shown.

#### Prevalence corrected for effect of method (with 95 % CI)

The trend curves show the previously reported increase in the estimated prevalence of depressive symptoms [26] in the adult population from around 2020 onwards. In addition, there is a significant jump between the last two estimates from the telephone surveys (blue) and the estimate for 2024 from the RKI Panel (grey): At 20.8 %, the prevalence estimate for 2024 is just under six percentage points above the estimate from GEDA 2024 for a largely overlapping period (June – September 2024; 15.1 % (13.1 % – 17.3 %)) and slightly more than six percentage points above the estimate for 2023 (14.4 % (13.3 % – 15.7 %)).

All grey (study type 1) and blue diamonds (study type 2) are close to the trend curves modelled for the respective study designs. This good fit suggests that the assumption of a constant effect of survey method is justified and that the panel estimate for 2024 (grey) is at the expected level given the observed development over time and the method used. The differences described between the most recent estimates are therefore likely mainly due to method effects [41], i.e. the effects of a change in sampling and a change in the survey mode on the prevalence estimates. This is particularly evident in the good fit of the estimates adapted to study type 1 (grey-bordered diamonds), which were collected in study type 2, to the grey trend curve.

The percentage point vertical difference between the grey and blue trend curves represents the magnitude of the effect of method, which was estimated for the total population as well as for gender, age and education groups (Table 3). For the total population, this effect is estimated at 4.2 percentage points with a confidence interval of 2.6 to 5.9 percentage points. This means that the prevalence of depressive symptoms in the RKI Panel 2024 is estimated to be about that much higher in a written survey in a registry sample than in a simultaneous telephone survey. Without taking confidence intervals into account, about four percentage points of the six-percentage-point difference between the 2023 and 2024 surveys are therefore estimated to be due to the change in method. Thus, a slight real increase in prevalence cannot be ruled out; however, given the statistical uncertainty of prevalence estimates and method effects, as well as the uncertainty of trend estimates at the margin, it also cannot be confirmed.

Method effects are particularly evident among women, in the high education group, and decrease with age. There may be no effects of method in the 80 + group (Table 3, Annex Table 10); however, statistical uncertainty is very high for this group. 18- to 29-year-olds stand out with an estimated prevalence difference of 11.2 percentage points between study type 1 and study type 2. The particularly large increase in depressive symptoms according to PHQ-8 ≥ 10 in this age group from 19.4 % (16.0 % – 23.5 %) in GEDA 2023 to 32.8 % (30.6 % – 35.1 %) in the RKI Panel 2024 could therefore be almost entirely attributable to methodological effects, as could increases between 2023 and 2024 in other groups. However, considerable statistical uncertainty prevents a conclusive assessment of changes between 2023 and 2024. When looking at the entire period from 2014/2015 to 2024, on the other hand, a clear increase in the prevalence of depressive symptoms can be observed across population groups (with some limitations for those aged 80 and above) (see corresponding indicator sheet in [41]).

The same pattern was found for anxiety symptoms (measured using the GAD-2 short screener), with higher prevalence estimates in registry samples with written surveys. However, models for this indicator are subject to greater uncertainty, as there are fewer survey points available for anxiety symptoms (only since 2022). Also, with the exception of the survey from the RKI Panel 2024, these only come from study type 2 (see indicator sheet method publication [41]).

## 4. Discussion

Data from the RKI Panel suggests that approximately one in four adults (25 %) in Germany exhibited symptoms of depression or anxiety in 2024, with around one third of this group (8 %) experiencing moderately severe to severe symptoms. Women, younger adults and those with low to medium levels of education were particularly affected. The highest prevalence was observed among young women aged 18 to 29, almost half of whom (47 %) showed symptoms of depression or anxiety. Notably, individuals with low educational attainment exhibit an almost threefold risk of more severe depressive symptoms and a twofold risk of more severe anxiety symptoms compared with those in the high level of education group.

Trend models using data from 2014 to 2024 suggests that prevalence estimates for 2024 are within the expected range taking into account the survey methodology used. The increases in prevalence observed between the 2023 telephone survey and the 2024 written survey (paper/online) with a registry sample are likely to be largely due to effects of change in methodology, which account for an estimated 4 percentage point difference in the prevalence of depressive symptoms among adults. A further slight deterioration in mental health following significant increases in symptoms until 2023 [26] can be neither confirmed nor ruled out. Possible effects of methods changes are particularly pronounced among 18- to 29-year-olds. They are also more pronounced among women than among men and in the high education group than in the other education groups.

###  

#### Prevalence and trends of depressive and anxiety symptoms in (inter)national comparison

Compared to previously published national and international findings, the prevalence rates reported here appear strikingly high. For example, the proportion of the population with depressive symptoms (PHQ-8 ≥ 10) measured using different survey modes in the European Health Interview Survey (EHIS) 2019/2020 in various European countries was between approximately 2 % and 11 % [47] (8.3 % in Germany, survey conducted as part of GEDA). For the years 2022 – 2024, some comparable results can be found: A non-representative but large-scale online study in three federal states reported rates of 16.0 % for anxiety (GAD-7 ≥ 10) and 16.4 % for depressive symptoms (PHQ-9 ≥ 10) for the summer of 2022 [48]. With regard to anxiety symptoms, these values are similar to the prevalence reported here for 2024 (14.3 %) and with regard to depressive symptoms, they correspond to the estimate from GEDA 2022 corrected for study type 1 (16.8 % with PHQ-8 ≥ 10). In the United Kingdom, a population-based survey with predominantly online participation for 2022 also showed a comparable prevalence of depressive symptoms of 16 % (PHQ-8 ≥ 10) and 28 % among 16- to 29-year-olds [31] (compared to 22.4 % among young adults aged 18 and over in Germany in 2022 according to the GEDA estimate corrected for study type 1). In the USA, a representative online survey found comparable or higher proportions for anxiety symptoms (GAD-2 ≥ 3) between approximately 17 % and 32 % (2022 – 2024) and for depressive symptoms (PHQ-2 ≥ 3) between 19 % and 25 % (2022 – 2023) [49].

As summarised elsewhere [26], the results of several comparable international studies show trend patterns different from the increase in depressive and anxiety symptoms observed in Germany between 2020 and 2023 [26–28]. However, in keeping with the results reported here, data from the German Socio-Economic Panel also indicate a decline in mental health-related quality of life (MHrQoL) among individuals under 50 years of age during the period from 2020 to 2022 [50]. Another German panel study using the same scale to assess HrQoL showed a near return to the pre-pandemic baseline by summer 2022, but a sustained decline in the psychological well-being subscale [49]. In addition, a renewed increase in the administrative prevalence of depression and anxiety disorders among adults with statutory health insurance in outpatient care was recorded for both 2023 and 2024 [21, 51], after diagnoses had previously developed differently from symptoms (see discussion of these discrepancies here [26]). In recent years, new record highs have been repeatedly reported in Germany for absence days from work due to mental disorders, most recently also for 2023 and 2024 [10, 53].

#### Assessment of the current mental health status and interpretation of trends

Due to the change in methodology and the statistical uncertainty of the trend modelling results presented above, it is not possible to assess whether the prevalence of depressive and anxiety symptoms increased further in 2024 compared to 2023. In particular for data points at the margins of the observation period, model-based approaches allow only a limited degree of accuracy [41]. The results of the GEDA survey conducted in parallel with the first two waves of the panel survey in the summer of 2024 suggest a stagnation in depressive symptoms; however, these findings are subject to considerable uncertainty due to the small sample size (Figure 1). Overall, therefore, developments in the mental health of the population from 2020 onwards remain concerning.

The data suggest persistently lower levels mental health of the population in recent years, but do not provide information on its causes. The interpretation of these findings must take into account a social context characterised by a polycrisis [54]. In addition to stressors related to the COVID-19 pandemic and intensifying climate crisis, the years from 2022 onwards saw economic recession and inflation with rising living costs [55] and a growing risk of poverty [56] as well as wars with an impact on life in Germany and extensive media coverage. Furthermore, it cannot be ruled out that survey participants’ response behaviour may have changed due to greater public awareness of mental health issues and growing health literacy, as well as a decline in the stigmatisation of mental disorders [57–60]. As a result, mental distress may now be reported more accurately and underreported to a lesser extent. Another possibility in debate is that cultural shifts are resulting in a misconstrual of mild distress as a mental health problem. However, it is unlikely that these developments have been so significant over the past five years that they alone can explain the observed trends. These trends were also not confined to an increase in mild cases [26], further suggesting that a rise in morbidity may have occurred.

#### Distribution of symptoms by gender

The finding that women are more likely to experience symptoms of depression and anxiety than men is consistent with previous results from health monitoring at the RKI and international literature (e.g. [15, 61–63]). Possible reasons for this gender difference include biological differences (e.g. neurophysiological and hormonal differences), effects of socio-cultural gender roles, and an unequal distribution of social stressors and stressful life events [14]. It is also conceivable that symptoms manifest differently in women and men and that depressive and anxiety symptoms in women are therefore better detected by common screening instruments [64].

#### Distribution of symptoms by age

The age distribution of symptom prevalence also follows the expected pattern: epidemiological studies show both a higher burden of depressive and anxiety symptoms and a higher prevalence of disorders (diagnosed in psychodiagnostic interviews) in younger adults [29, 61, 65–67]. The transition to adulthood is a phase of life marked by profound changes, characterised by leaving the parental home, finding one’s identity, starting a career, starting a family and building financial independence [68]. This increase in responsibility can be accompanied by increased stress [69]. Since most mental disorders first appear before the age of 25 [70], this phase of life is considered particularly critical for mental health.

Internationally [69, 71, 72] and in Germany [73], there is evidence that the mental health of younger adults compared to older adults has deteriorated across cohorts and that depressive symptoms have become more common [73]. The reasons discussed include increasing psychosocial risks such as stress, social isolation and intensive use of social media [73] in addition to worldwide stressors such as climate change, economic uncertainty and financial pressures [71], as well as differences in the perception of symptom severity between generations [73]. The multiple crises of recent years may have placed additional strain on this age group, as worries may be more strongly associated with mental health among younger people [74]. Specific stresses in the aftermath of the COVID-19 pandemic may also have contributed, including persistent loneliness in this age group [75] and the particular impact of economic uncertainty during the transition into tertiary education and working life. Women aged 18 to 29 already stood out with high levels of depressive systems in data from the GEDA telephone survey 2023 [26]. The results for 2024 underscore the risk group status of young women and the urgent need for a better understanding of their specific stressors, as well as measures to promote the mental health of girls and young women.

Administrative prevalence rates for depression and anxiety disorders generally show a different age distribution than symptoms in the general population. However, these indicators also showed notable results for 18- to 29-year-olds in recent years, with diagnoses rising comparatively sharply in this group between 2021 and 2023 [21, 52]. This may indicate both an increase in morbidity and a change in healthcare utilisation, and further underscores the need for heightened attention to mental health developments in this group. According to a report by the Organisation for Economic Co-operation and Development (OECD) which includes a best practice example from Germany, most countries report that they have developed new mental health prevention programmes for children and young adults during or after the COVID-19 pandemic. However, all countries are found to have policy gaps, gaps in the implementation of measures, and high levels of unmet need [76].

#### Distribution of symptoms by education

The finding of a higher prevalence of depressive and anxiety symptoms in the low and medium education groups compared to the high education group is consistent with the general evidence [77]. Whether the increases in socioeconomic inequalities in the prevalence of depressive symptoms observed for 2022 and 2023 using GEDA data [32] continued in 2024 cannot be assessed due to the change in methodology. In view of the considerable social inequality in these health indicators, preventive approaches addressing structural and systemic determinants as well as mental health in all policies strategies [78] are of particular importance. In view of the interaction between gender and education reported here, possible gender-specific distribution patterns should also be taken into account – in particular, the symptom burden among men in the low education group. This was already evident with data from 2023, which showed a high prevalence of depressive symptoms and a noticeable increase compared to 2022 in this group against a backdrop of increasing financial strain [26]. It is important to note that women in the low education group are not less affected; instead, the low education group does not exhibit the gender differences in symptom prevalence seen in the high and medium education groups.

#### Effects of changes in survey methodology

The effects of methods changes described, which result in higher estimated prevalences for study type 1 (studies with a registry sample and a written mixed-mode design, e.g. RKI Panel 2024) compared to study type 2 (studies based on a telephone sample and telephone interviews), are examined in depth in another article [41]. According to this work, the observed effects are consistent with the literature, especially with regard to instruments that capture subjective assessments using Likert-like response scales [79]. The three most important proposed explanations for these methodological effects suggest that the methodology used in the RKI Panel likely captures the mental health status of the population better than a telephone survey:

1. Firstly, it has been established that self-reports in written and online surveys are less affected by social desirability effects and are more honest with regard to perceived stress than self-reports to an interviewer [80].
2. Secondly, inventories with multiple, sometimes complex response categories (e.g. ‘more than half the days’ for PHQ and GAD) may be easier for participants to process and answer in writing than in a telephone interview. As reported in the article examining the change in methodology [41], a comparison of study type 1 with study type 2 shows a shift from the first and final response categories (‘not at all’ and ‘nearly every day’) to a more frequent use of the middle response categories (‘several days’, ‘more than half the days’), resulting in higher sum scores and thus higher prevalence estimates given that the first and last categories are unevenly filled. This could indicate a more differentiated response to these screening instrument items in the RKI Panel 2024. Alternatively, or in part, this may also be due to well-known central tendency effects found for scales in written surveys [79].
3. Thirdly, samples in study type 1 better reflect the population structure in Germany than samples in study type 2 [41]. In particular, the RKI Panel reaches several hard-to-reach groups more effectively than a telephone survey, including young adults, older adults, and those with lower educational attainment. The particularly pronounced methodological effects among 18- to 29-year-olds could therefore be attributed to both a change in response behaviour and a change in the composition of the sample, which may be more representative in the RKI Panel. At the same time, the multi-phase participation process (registration, active participation in repeated surveys, completing the mental health questionnaire) may result in selective participation by certain individuals within this subgroup. It is conceivable that individuals with mental health problems are more likely to participate in health surveys in general and, in particular, in mental health surveys. This could contribute to an overestimation of prevalence. Evidence on this particular selection bias is mixed. For example, in a mental health survey conducted within a self-recruiting online panel, the proportion of participants with mental health problems was 2.5 times higher than among non-participants, despite the use of stratified random sampling for this study [81]. In contrast, a survey study involving the RKI among members of a large German health insurance company found lower administrative prevalence of affective disorders among participants [82]. Differences in methodological effects between population groups should therefore be investigated more systematically in future. This conclusion is also reached by a study which, in line with the present results, reported higher estimated levels of depressive symptoms in young adults and highly educated individuals in written self-reports than in face-to-face interviews [83].

Overall, it can be said that estimated prevalence must always be assessed against the background of the employed survey methodology. There is evidence that psychometric instruments [84, 85], including the PHQ-8 [84], are measurement-invariant across survey modes: while mean values may differ between modes, the latent constructs measured appear to remain consistent. In the case of screening instruments, the validity of established cutoffs should also be assessed across different survey methods. The thresholds for PHQ and GAD have, however, been repeatedly validated using written self-administered questionnaires (see studies in meta-analyses [43, 86]), which further speaks to the reliability of the prevalence estimates for 2024.

#### Strengths and limitations

Several strengths and limitations should be considered when interpreting the results of this study. One notable strength is the substantial sample size of the RKI Panel. Recruitment via registry offices and a study-specific weighting factor ensure a high degree of representativeness for the adult population living in Germany. In addition to health and sociodemographics, the survey also collects information on other topics that can be incorporated into analyses. Nevertheless, certain distortions – such as selective non-participation (selection bias) – cannot be fully ruled out. Individuals who are interested in health topics and health-conscious behaviour may be more likely to participate. Unregistered individuals (e.g., people experiencing homelessness) are, by design, excluded from the sampling frame, and persons residing in institutions such as nursing homes are likewise generally not included. Another limitation is the requirement of good written German for participation. Trend modelling aimed at contextualising the RKI Panel 2024 results within existing time series and assessing the effects of methodological changes is constrained by the limited number of data points – particularly the few observations based on registry samples with written surveys. Moreover, model-based estimates can approximate data points at the margins of the observation period (in this case, the estimates for 2024) only to a limited extent. Continued observation is therefore required to enable a reliable assessment of temporal trends. Finally, it should be noted that screening instruments do not yield clinical diagnoses.

#### Conclusion

The estimated prevalence of depressive and anxiety symptoms among adults in Germany in 2024 is considerable in magnitude. The symptom burden has increased significantly in recent years against a backdrop of multiple collective crises. The findings underscore the need for measures to promote and protect mental health. In particular, the needs of young adults, and especially young women, should be addressed. Given the social inequality in these health indicators, preventive approaches targeting structural and systemic determinants are of central importance. The establishment of the RKI Panel will enable a methodologically consistent observation of depressive and anxiety symptoms over time, fulfilling a key prerequisite for the reliable assessment of trends in the population.

## Figures and Tables

**Table 1:** Prevalence of depressive and anxiety symptoms in adults in Germany in 2024 (weighted in %, with 95 % confidence intervals). Source: RKI Panel 2024

	Depressive/anxiety symptoms						More severe depressive/anxiety symptoms					
	PHQ-9 ≥ 10		GAD-7 ≥ 10		PHQ-9 or GAD-7 ≥ 10		PHQ-9 ≥ 15		GAD-7 ≥ 15		PHQ-9 or GAD-7 ≥ 15	
	%	(95 % CI)	%	(95 % CI)	%	(95 % CI)	%	(95 % CI)	%	(95 % CI)	%	(95 % CI)
Total	21.9	(21.1 – 22.7)	14.3	(13.6 – 15.0)	25.1	(24.3 – 26.0)	7.1	(6.6 – 7.6)	4.1	(3.7 – 4.4)	8.4	(7.9 – 9.0)
Women	24.4	(23.3 – 25.5)	16.5	(15.6 – 17.5)	28.0	(26.9 – 29.2)	8.1	(7.4 – 8.8)	5.2	(4.7 – 5.8)	9.8	(9.0 – 10.6)
Men	19.0	(18.0 – 20.1)	11.9	(11.0 – 12.8)	21.9	(20.8 – 23.1)	5.9	(5.3 – 6.6)	2.8	(2.4 – 3.3)	6.9	(6.2 – 7.6)
18 – 29 years	34.2	(32.1 – 36.5)	23.6	(21.7 – 25.6)	38.9	(36.6 – 41.2)	12.4	(10.8 – 14.2)	6.6	(5.6 – 7.8)	14.0	(12.4 – 15.9)
30 – 44 years	23.5	(21.8 – 25.3)	17.2	(15.8 – 18.7)	27.7	(25.9 – 29.5)	7.7	(6.8 – 8.8)	4.9	(4.2 – 5.8)	9.6	(8.6 – 10.8)
45 – 64 years	20.5	(19.2 – 21.7)	13.1	(12.1 – 14.2)	23.3	(22.0 – 24.6)	6.6	(5.8 – 7.4)	4.0	(3.4 – 4.6)	7.7	(6.9 – 8.6)
65 – 79 years	12.2	(11.1 – 13.4)	6.7	(5.9 – 7.6)	14.4	(13.3 – 15.7)	2.8	(2.3 – 3.4)	1.3	(0.9 – 1.7)	3.3	(2.8 – 4.0)
80 + years	20.1	(17.5 – 22.9)	9.5	(7.6 – 11.8)	21.8	(19.2 – 24.7)	6.2	(4.7 – 8.1)	3.2	(2.2 – 4.6)	7.8	(6.0 – 10.2)
Low education	24.9	(23.2 – 26.7)	15.1	(13.6 – 16.7)	27.5	(25.8 – 29.4)	8.6	(7.5 – 9.9)	4.7	(3.9 – 5.6)	9.9	(8.7 – 11.2)
Medium education	22.6	(21.7 – 23.7)	15.0	(14.1 – 15.8)	26.1	(25.1 – 27.2)	7.4	(6.8 – 8.0)	4.1	(3.6 – 4.6)	8.7	(8.1 – 9.4)
High education	15.4	(14.4 – 16.4)	11.4	(10.5 – 12.4)	19.0	(18.0 – 20.2)	3.9	(3.4 – 4.5)	3.0	(2.5 – 3.5)	5.3	(4.7 – 5.9)

**Table 2:** Prevalence of depressive and anxiety symptoms among adults in Germany in 2024 by gender and age, gender and education (weighted in %, with 95 % confidence intervals). Source: RKI Panel 2024

	Depressive/anxiety symptoms												More severe depressive/anxiety symptoms											
	PHQ-9 ≥ 10				GAD-7 ≥ 10				PHQ-9 or GAD-7 ≥ 10				PHQ-9 ≥ 15				GAD-7 ≥ 15				PHQ-9 or GAD-7 ≥ 15			
	Women		Men		Women		Men		Women		Men		Women		Men		Women		Men		Women		Men	
	%	(95 % CI)	%	(95 % CI)	%	(95 % CI)	%	(95 % CI)	%	(95 % CI)	%	(95 % CI)	%	(95 % CI)	%	(95 % CI)	%	(95 % CI)	%	(95 % CI)	%	(95 % CI)	%	(95 % CI)
18 – 29 years	41.7	(38.7 – 44.8)	27.0	(24.0 – 30.3)	29.4	(26.6 – 32.4)	18.1	(15.4 – 21.2)	47.0	(43.9 – 50.1)	31.2	(27.8 – 34.8)	16.1	(13.7 – 18.8)	8.6	(6.7 – 11.0)	9.4	(7.7 – 11.4)	3.9	(2.8 – 5.3)	18.3	(15.8 – 21.0)	9.6	(7.7 – 12.0)
30 – 44 years	26.6	(24.4 – 28.9)	20.3	(18.1 – 22.8)	20.9	(18.9 – 23.0)	13.6	(11.7 – 15.7)	31.3	(29.0 – 33.6)	24.0	(21.7 – 26.5)	9.0	(7.7 – 10.5)	6.5	(5.2 – 8.3)	6.9	(5.8 – 8.3)	3.0	(2.2 – 4.0)	11.7	(10.3 – 13.3)	7.6	(6.2 – 9.4)
45 – 64 years	22.5	(20.9 – 24.2)	18.3	(16.6 – 20.2)	14.7	(13.4 – 16.2)	11.5	(10.0 – 13.1)	25.6	(23.9 – 27.4)	20.9	(19.1 – 22.8)	6.9	(6.0 – 8.0)	6.3	(5.3 – 7.4)	4.6	(3.8 – 5.5)	3.4	(2.6 – 4.3)	8.1	(7.1 – 9.3)	7.3	(6.2 – 8.6)
65 – 79 years	12.9	(11.4 – 14.5)	11.5	(10.0 – 13.2)	7.7	(6.5 – 9.1)	5.7	(4.6 – 6.9)	15.5	(13.9 – 17.3)	13.2	(11.7 – 15.0)	3.1	(2.4 – 4.0)	2.4	(1.7 – 3.3)	1.7	(1.1 – 2.4)	0.8	(0.4 – 1.5)	3.9	(3.1 – 4.9)	2.7	(2.0 – 3.7)
80 + years	22.2	(18.6 – 26.3)	16.8	(13.7 – 20.5)	10.5	(7.9 – 13.9)	8.0	(5.8 – 10.8)	24.1	(20.4 – 28.3)	18.4	(15.2 – 22.1)	7.5	(5.3 – 10.6)	4.1	(2.7 – 6.3)	4.2	(2.8 – 6.4)	1.7	(0.8 – 3.6)	9.5	(6.8 – 13.2)	5.3	(3.5 – 7.8)
Low education	25.8	(23.4 – 28.3)	23.8	(21.5 – 26.3)	15.9	(13.9 – 18.2)	14.2	(12.3 – 16.4)	28.5	(26.1 – 31.1)	26.4	(23.9 – 29.0)	9.8	(8.0 – 11.8)	7.4	(6.2 – 8.9)	5.7	(4.6 – 7.2)	3.6	(2.7 – 4.8)	11.3	(9.4 – 13.4)	8.5	(7.2 – 10.1)
Medium education	25.6	(24.3 – 26.9)	19.1	(17.7 – 20.5)	17.6	(16.4 – 18.8)	11.9	(10.8 – 13.0)	29.3	(28.0 – 30.7)	22.2	(20.8 – 23.8)	8.2	(7.5 – 9.1)	6.2	(5.4 – 7.2)	5.2	(4.6 – 5.9)	2.7	(2.2 – 3.3)	10.0	(9.1 – 10.9)	7.1	(6.2 – 8.1)
High education	19.0	(17.5 – 20.7)	12.2	(11.0 – 13.5)	14.8	(13.5 – 16.3)	8.5	(7.3 – 9.8)	23.8	(22.1 – 25.4)	15.0	(13.6 – 16.4)	4.6	(3.9 – 5.5)	3.2	(2.6 – 4.0)	4.3	(3.6 – 5.2)	1.8	(1.3 – 2.3)	6.6	(5.7 – 7.6)	4.1	(3.4 – 4.9)

**Table 3:** Estimated effect of the methodological difference between study type 1 and study type 2 on the estimated prevalence of depressive symptoms (PHQ-8 ≥ 10) in percentage points. Source: GEDA 2014/2015-EHIS, GEDA 2019/2020-EHIS, GEDA 2022, GEDA 2023, GEDA 2024, RKI Panel 2024

	Percentage points	(95 % CI)	p-value
Total	4.2	(2.6 – 5.9)	p < 0.0001
Women	6.0	(3.7 – 8.3)	p^*^ = 0 0225
Men	2.2	(- 0.1 – 4.5)	
18 – 29 years	11.2	(6.4 – 16.0)	p^*^ = 0.0098
30 – 44 years	4.4	(0.7 – 8.0)	
45 – 64 years	2.6	(- 0.0 – 5.2)	
65 – 79 years	2.6	(1.2 – 4.0)	
80 + years	-0.1	(- 5.7 – 5.6)	
Low education	3.7	(- 1.5 – 8.9)	p^*^ = 0.0353
Medium education	3.6	(1.5 – 5.7)	
High education	6.6	(5.5 – 7.7)	

Method effect expressed as the difference in the modelled time trend for the two study types 1 (EMA sample and written/online survey; RKI Panel 2024 and GEDA 2014/2015) and 2 (telephone surveys). Method effect > 0 indicates that the prevalence estimates are higher in study type 1.

^*^p-values for group differences from Wald tests

**Annex Table 1:** Sample Characteristics RKI Panel 2024 – Questionnaire C

Group	n	% weighted
Total n	27,102	
Gender female	14,709	51.1 %
Gender male	12,329	48.9 %
Age group 18 – 29 years	3,829	15.9 %
Age group 30 – 44 years	5,761	23.5 %
Age group 45 – 64 years	9,025	33.4 %
Age group 65 – 79 years	6,201	18.5 %
Age group 80 + years	2,286	8.7 %
Low education	5,201	33.3 %
Medium education	12,964	46.4 %
High education	8,888	20.3 %

**Annex Table 2:** Depressive symptoms (PHQ-9 ≥ 10) regressed on age, gender and education (Poisson regression). Source: RKI Panel 2024

Predictor	Category/level	RR	(95 % CI)	t	p-value
Gender	Women (Ref.: Men)	1.32	(1.24 – 1.41)	8.49	< 0.001
Age	18 – 29 years (Ref.: 45 – 64)	1.72	(1.58 – 1.88)	12.47	< 0.001
Age	30 – 44 years (Ref.: 45 – 64)	1.22	(1.11 – 1.34)	4.25	< 0.001
Age	65 – 79 years (Ref.: 45 – 64)	0.56	(0.50 – 0.62)	- 10.61	< 0.001
Age	80 + years (Ref.: 45 – 64)	0.83	(0.72 – 0.96)	- 2.44	0.015
Education	Low education (Ref.: High)	1.84	(1.66 – 2.04)	11.84	< 0.001
Education	Medium education (Ref.: High)	1.38	(1.28 – 1.49)	8.44	< 0.001
Education^*^	Medium education (Ref.: Low)	0.75	(0.69 – 0.82)	- 6.64	< 0.001

^*^Education: results additionally shown with low education group as reference for testing differences between low and medium education groups. RR = relative risk. RR > 1 higher risk relative to reference; RR < 1 lower risk relative to reference. Weighted analysis.

**Annex Table 3:** Depressive symptoms (PHQ-9 ≥ 10) regressed on age, gender and education (Poisson regression), with gender × age interaction. Source: RKI Panel 2024

Predictor	Category/level	RR	(95 % CI)	t	p-value
Gender	Women (Ref.: Men)	1.23	(1.10 – 1.38)	3.55	< 0.001
Age	18 – 29 years (Ref.: 45 – 64)	1.50	(1.29 – 1.76)	5.12	< 0.001
Age	30 – 44 years (Ref.: 45 – 64)	1.16	(1.00 – 1.35)	1.98	0.049
Age	65 – 79 years (Ref.: 45 – 64)	0.61	(0.52 – 0.71)	- 6.18	< 0.001
Age	80 + years (Ref.: 45 – 64)	0.84	(0.67 – 1.06)	- 1.48	0.140
Education	Low education (Ref.: High)	1.86	(1.68 – 2.05)	12.02	< 0.001
Education	Medium education (Ref.: High)	1.39	(1.29 – 1.50)	8.64	< 0.001
Interaction: Gender × Age	Women × 18 – 29 years	1.27	(1.05 – 1.54)	2.50	0.013
Interaction: Gender × Age	Women × 30 – 44 years	1.10	(0.92 – 1.31)	1.02	0.307
Interaction: Gender × Age	Women × 65 – 79 years	0.87	(0.71 – 1.06)	- 1.37	0.172
Interaction: Gender × Age	Women × 80 + years	0.99	(0.74 – 1.33)	- 0.07	0.948

RR = relative risk. RR > 1 higher risk relative to reference; RR < 1 lower risk relative to reference. Weighted analysis. Wald test result for interaction gender x age: p = 0.0114

**Annex Table 4:** Depressive symptoms (PHQ-9 ≥ 10) regressed on age, gender and education (Poisson regression), with interaction between gender and education. Source: RKI Panel 2024

Predictor	Category/level	RR	(95 % CI)	t	p-value
Gender	Women (Ref.: Men)	1.48	(1.30 – 1.69)	5.88	< 0.001
Age	18 – 29 years (Ref.: 45 – 64)	1.72	(1.58 – 1.88)	12.47	< 0.001
Age	30 – 44 years (Ref.: 45 – 64)	1.22	(1.11 – 1.34)	4.20	< 0.001
Age	65 – 79 years (Ref.: 45 – 64)	0.56	(0.50 – 0.63)	- 10.39	< 0.001
Age	80 + years (Ref.: 45 – 64)	0.85	(0.73 – 0.98)	- 2.24	0.026
Education	Low education (Ref.: High)	2.09	(1.81 – 2.42)	9.99	< 0.001
Education	Medium education (Ref.: High)	1.42	(1.26 – 1.61)	5.63	< 0.001
Interaction: Gender × Education	Women × Low education	0.79	(0.66 – 0.95)	- 2.47	0.014
Interaction: Gender × Education	Women × Medium education	0.94	(0.81 – 1.10)	- 0.78	0.439

RR = relative risk. RR > 1 higher risk relative to the reference; RR < 1 lower risk relative to the reference. Weighted analysis. Wald test result for interaction gender × education: p = 0.0352

**Annex Table 5:** More severe depressive symptoms (PHQ-9 ≥ 15) regressed on age, gender and education (Poisson regression). Source: RKI Panel 2024

Predictor	Category/level	RR	(95 % CI)	t	p-value
Gender	Women (Ref.: Men)	1.42	(1.24 – 1.63)	5.13	< 0.001
Age	18 – 29 years (Ref.: 45 – 64)	1.95	(1.63 – 2.34)	7.26	< 0.001
Age	30 – 44 years (Ref.: 45 – 64)	1.30	(1.09 – 1.56)	2.91	0.004
Age	65 – 79 years (Ref.: 45 – 64)	0.38	(0.30 – 0.48)	- 7.82	< 0.001
Age	80 + years (Ref.: 45 – 64)	0.73	(0.54 – 0.99)	- 2.07	0.039
Education	Low education (Ref.: Hgh)	2.70	(2.19 – 3.33)	9.38	< 0.001
Education	Medium education (Ref.: Hgh)	1.75	(1.49 – 2.06)	6.83	< 0.001
Education^*^	Medium education (Ref.: Low)	0.65	(0.55 – 0.77)	- 5.03	< 0.001

^*^Education: results additionally shown with low education group as reference for testing differences between low and medium education groups. RR = relative risk. RR > 1 higher risk relative to reference; RR < 1 lower risk relative to reference. Weighted analysis.

**Annex Table 6:** More severe depressive symptoms (PHQ-9 ≥ 15) regressed on age, gender and education (Poisson regression), with gender × age interaction. Source: RKI Panel 2024

Predictor	Category/level	RR	(95 % CI)	t	p-value
Gender	Women (Ref.: Men)	1.10	(0.89 – 1.37)	0.91	0.364
Age	18 – 29 years (Ref.: 45 – 64)	1.42	(1.05 – 1.92)	2.29	0.023
Age	30 – 44 years (Ref.: 45 – 64)	1.12	(0.83 – 1.51)	0.76	0.450
Age	65 – 79 years (Ref.: 45 – 64)	0.36	(0.25 – 0.52)	- 5.48	< 0.001
Age	80 + years (Ref.: 45 – 64)	0.58	(0.36 – 0.93)	- 2.28	0.023
Education	Low education (Ref.: High)	2.72	(2.21 – 3.35)	9.44	< 0.001
Education	Medium education (Ref.: High)	1.77	(1.50 – 2.08)	6.90	< 0.001
Interaction: Gender × Age	Women × 18 – 29 years	1.74	(1.22 – 2.47)	3.07	0.002
Interaction: Gender × Age	Women × 30 – 44 years	1.31	(0.914 – 1.88)	1.47	0.142
Interaction: Gender × Age	Women × 65 – 79 years	1.09	(0.69 – 1.72)	0.37	0.709
Interaction: Gender × Ager	Women × 80 + years	1.47	(0.81 – 2.68)	1.26	0.208

RR = Relative risk. RR > 1 higher risk relative to the reference; RR < 1 lower risk relative to the reference. Weighted analysis. Wald test result for interaction gender x age: p = 0.0393

**Annex Table 7:** Anxiety symptoms (GAD-7 ≥ 10) regressed on age, gender and education (Poisson regression). Source: RKI Panel 2024

Predictor	Category/level	RR	(95 % CI)	t	p-value
Gender	Women (Ref.: Men)	1.46	(1.33 – 1.60)	8.00	< 0.001
Age	18 – 29 years (Ref.: 45 – 64)	1.85	(1.65 – 2.08)	10.51	< 0.001
Age	30 – 44 years (Ref.: 45 – 64)	1.38	(1.23 – 1.54)	5.55	< 0.001
Age	65 – 79 years (Ref.: 45 – 64)	0.48	(0.41 – 0.56)	- 9.25	< 0.001
Age	80 + years (Ref.: 45 – 64)	0.61	(0.49 – 0.77)	- 4.26	< 0.001
Education	Low education (Ref.: Hgh)	1.63	(1.43 – 1.85)	7.33	< 0.001
Education	Medium education (Ref.: Hgh)	1.23	(1.12 – 1.35)	4.24	< 0.001
Education^*^	Medium education (Ref.: Low)	0.76	(0.67 – 0.85)	- 4.69	< 0.001

^*^Education: results additionally shown with low education group as reference for testing differences between low and medium education groups. RR = relative risk. RR > 1 higher risk relative to reference; RR < 1 lower risk relative to reference. Weighted analysis.

**Annex Table 8:** More severe anxiety symptoms (GAD-7 ≥ 15) regressed on age, gender and education (Poisson regression). Source: RKI Panel 2024

Predictor	Category/level	RR	(95 % CI)	t	p-value
Gender	Women (Ref.: Men)	1.98	(1.66 – 2.37)	7.52	< 0.001
Age	18 – 29 years (Ref.: 45 – 64)	1.75	(1.41 – 2.18)	5.01	< 0.001
Age	30 – 44 years (Ref.: 45 – 64)	1.34	(1.06 – 1.69)	2.47	0.014
Age	65 – 79 years (Ref.: 45 – 64)	0.28	(0.19 – 0.40)	- 6.84	< 0.001
Age	80 + years (Ref.: 45 – 64)	0.63	(0.42 – 0.93)	- 2.32	0.021
Education	Low education (Ref.: High)	2.01	(1.56 – 2.60)	5.37	< 0.001
Education	Medium education (Ref.: High)	1.28	(1.04 – 1.56)	2.40	0.017
Education^*^	Medium education (Ref.: Low)	0.63	(0.51 – 0.79)	- 4.08	< 0.001

^*^Education: results additionally shown with low education group as reference for testing differences between low and medium education groups. RR = relative risk. RR > 1 higher risk relative to reference; RR < 1 lower risk relative to reference. Weighted analysis.

**Annex Table 9:** Prevalence of depressive symptoms (according to PHQ-8) among adults in Germany in 2024 (weighted in %, with 95 % confidence intervals (CI)). Source: RKI Panel 2024

	PHQ-8 ≥ 10	(95 % KI)	PHQ-8 ≥ 15	(95 % CI)
Total	20.8	(20.0 – 21.6)	5.9	(5.4 – 6.3)
Women	23.6	(22.5 – 24.7)	7.0	(6.3 – 7.7)
Men	17.8	(16.8 – 18.8)	4.7	(4.1 – 5.3)
18 – 29 years	32.4	(30.2 – 34.7)	10.0	(8.5 – 11.7)
30 – 44 years	22.8	(21.1 – 24.5)	6.1	(5.3 – 7.1)
45 – 64 years	19.6	(18.4 – 20.9)	5.9	(5.2 – 6.7)
65 – 79 years	11.3	(10.2 – 12.4)	2.3	(1.8 – 2.8)
80 + years	18.3	(15.8 – 21.1)	4.9	(3.6 – 6.7)
Low education	23.6	(22.0 – 25.4)	7.2	(6.2 – 8.4)
Medium education	21.5	(20.5 – 22.5)	6.1	(5.5 – 6.6)
High education	14.7	(13.7 – 15.7)	3.2	(2.7 – 3.7)

**Annex Table 10:** Prevalence of depressive symptoms (according to PHQ-8) among adults in Germany in 2023 and 2024 (weighted in %, with 95 % confidence intervals (CI)). Source: GEDA 2023 and RKI Panel 2024

	GEDA 2023 PHQ-8 ≥ 10	(95 % KI)	RKI-Panel 2024 PHQ-8 ≥ 10	(95 % CI)
Total	14.4	(13.3 – 15.7)	20.8	(20.0 – 21.6)
Women	15.4	(13.9 – 17.1)	23.6	(22.5 – 24.7)
Men	13.2	(11.6 – 15.0)	17.8	(16.8 – 18.8)
18 – 29 years	19.4	(16.0 – 23.5)	32.4	(30.2 – 34.7)
30 – 44 years	15.2	(12.7 – 18.1)	22.8	(21.1 – 24.5)
45 – 64 years	15.5	(13.6 – 17.5)	19.6	(18.4 – 20.9)
65 – 79 years	8.3	(6.8 – 10.1)	11.3	(10.2 – 12.4)
80 + years	11.4	(8.9 – 14.3)	18.3	(15.8 – 21.1)
Low education	20.5	(17.7 – 23.6)	23.6	(22.0 – 25.4)
Medium education	14.4	(12.9 – 16.0)	21.5	(20.5 – 22.5)
High education	7.0	(5.9 – 8.2)	14.7	(13.7 – 15.7)
